# Explainable Machine Learning for Concurrent Screening of Elevated Work-Related Fatigue Using Routine Working-Hour and Occupational Health Data Among Manufacturing Workers: A Cross-Sectional Model Development and Internal Validation Study

**DOI:** 10.3390/healthcare14162627

**Published:** 2026-08-19

**Authors:** Chien-Chih Wang, Ming-Shu Chen

**Affiliations:** 1 Department of Industrial Engineering and Management, Ming Chi University of Technology, New Taipei City 243303, Taiwan; 2 Department of Healthcare Administration, College of Healthcare & Management, Asia Eastern University of Science and Technology, New Taipei City 220303, Taiwan; tree@mail.aeust.edu.tw

**Keywords:** work-related fatigue, cumulative overtime, occupational health examination, occupational health surveillance, explainable machine learning, human-in-the-loop decision support

## Abstract

**Highlights:**

**What are the main findings?**

- Medium-term cumulative overtime and routinely measured occupational health indicators were key contributors to the model-estimated probability of elevated work-related fatigue among manufacturing workers in this study.
- In internal validation, XGBoost showed significantly higher discrimination than logistic regression (paired AUC difference, 0.09; *p* < 0.01), but its probability estimates were miscalibrated, and the study-specific review-priority thresholds remained provisional pending independent validation.

**What are the implications of the main findings?**

- Routine working-hour records and occupational health examination data can be transformed into auditable concurrent screening signals to support preventive overtime reviews.
- The proposed review-priority tiers illustrate a potential framework for differentiated occupational health reviews but should not be interpreted as implementation-ready decision rules.

**Abstract:**

**Background:** Work-related fatigue is a preventable occupational health concern in high-intensity manufacturing environments. However, supervisors and occupational health services often lack transparent criteria to support the preventive review of overtime practices. This study developed and internally validated an explainable human-in-the-loop concurrent screening model to identify employees with currently elevated work-related fatigue using routinely collected working-hour records and occupational health examination data. **Materials and Methods:** De-identified administrative and occupational health data from 314 full-time manufacturing employees were linked with standardized fatigue assessment data. Currently elevated work-related fatigue was defined using the top quartile of the standardized work-related fatigue subscale. To reduce information leakage, the work-related fatigue score used to define the outcome was excluded from the predictor sets. Multivariable logistic regression and XGBoost were compared using nested stratified five-fold cross-validation, with aggregated out-of-fold predictions from the outer folds used for the performance evaluation. Model performance was assessed using discrimination, probabilistic accuracy, calibration, and classification metrics at provisional, study-specific thresholds. **Results:** XGBoost demonstrated greater discrimination and better overall probabilistic accuracy than logistic regression, with an AUC of 0.77 versus 0.68 and a Brier score of 0.14 versus 0.17, respectively. At the provisional study-specific threshold of τ1 = 0.28, XGBoost achieved a sensitivity of 0.78 and a specificity of 0.70. Using τ1 = 0.28 and τ2 = 0.55, 58.0%, 27.1%, and 15.0% of employees were classified into provisional low-, moderate-, and high-priority tiers, respectively. The observed prevalence of currently elevated work-related fatigue increased across these tiers from 9.3% to 28.2% and 78.7%, respectively. Calibration assessment indicated systematic miscalibration for both XGBoost (intercept α = 0.78, slope β = 1.64) and logistic regression (α = −0.49, β = 0.56). Because τ1 and τ2 were selected and evaluated using aggregated out-of-fold predictions generated from the same single-organization development dataset, their stability and transportability are unknown. Medium-term cumulative overtime, particularly during months −2 to −6, together with routinely measured indicators of vulnerability and recovery, contributed substantially to the model-estimated probability of currently elevated fatigue. **Conclusions:** Routinely collected working-hour and occupational health examination data may support explainable concurrent screening for currently elevated work-related fatigue and illustrate a potential framework for differentiated preventive reviews. However, the proposed thresholds and review-priority tiers are provisional and study-specific and should not be interpreted as implementation-ready decision rules for occupational health practices. Prospective temporal validation, external validation in independent organizations, and local recalibration are required before threshold-based implementation.

## 1. Introduction

High-intensity manufacturing workplaces increasingly depend on overtime to sustain productivity, production flexibility, and global competitiveness. Rapid digitalization, compressed production schedules, and pressure from global supply chains have heightened work demands, making extended working hours a persistent challenge for occupational health services and workplace management. In many settings, overtime is financially incentivized through premium pay and wage–hour contracting, which may further normalize prolonged working hours [1,2]. From a preventive healthcare perspective, the central question is not only whether long working hours are harmful but also how organizations can determine when continued overtime work should prompt timely, transparent, and proportionate health reviews.

Substantial epidemiological evidence links prolonged working hours and insufficient recovery to adverse health and safety outcomes, including cardiovascular morbidity and mortality, across diverse occupational groups [3,4,5,6]. A joint analysis by the World Health Organization and the International Labour Organization estimated that exposure to working 55 or more hours per week was associated with 745,000 deaths worldwide in 2016, primarily from ischemic heart disease and stroke [6]. Similar concerns have been reported in shift work and high-risk occupations, including nursing, where safe limits for working hours remain an important occupational health issue [7]. Despite this evidence, many workplaces continue to rely on fixed overtime thresholds or ad hoc managerial judgments. These approaches may be difficult to audit and may not sufficiently account for cumulative exposure, recovery deficits, or individual vulnerability.

Overwork-related harm is generally understood to be a cumulative process. Sustained job demands reduce opportunities for recovery and may disrupt physiological, cognitive, and psychosocial regulations. From the perspective of occupational health psychology, this pattern reflects an imbalance between job demands and recovery opportunities, whereby repeated insufficient recovery contributes to the accumulation of psychophysiological strain. Within this framework, work-related fatigue is not simply transient. It may serve as a proximal and potentially modifiable indicator of strain that precedes more severe health or safety issues. Fatigue can impair safety-critical functions, including vigilance, decision-making, and error control, and is therefore relevant to both worker health and organizational risk management [8]. In high-intensity environments, inadequate recovery may contribute to sleep restriction, circadian disruption, and biological dysregulation [9]. Psychosocial stressors and overcommitment may further increase the vulnerability of workers exposed to sustained workload pressure [9,10].

Occupational fatigue is a multidimensional condition involving physical and cognitive exhaustion, reduced alertness, and impaired functional capacity [11,12,13,14]. However, in routine occupational health practice, fatigue assessment is often fragmented, reactive, or dependent on self-report instruments that are not continuously available for operational decision-making. Reviews of fatigue assessment tools have identified limitations in their accuracy, applicability, and contextual transferability across occupational settings [15]. Fatigue risk management systems can support prevention; however, their organizational implementation often remains incomplete, with limited integration of working-hour records, occupational health examination data, and preventive governance actions [16]. These limitations create a practical gap: occupational health professionals and supervisors need transparent, auditable, and prevention-oriented tools to identify workers who may require additional review before continued overtime work is approved.

Routinely collected workplace and occupational health data provide a feasible infrastructure for decision support. Administrative working-hour records can quantify both short-term and cumulative overtime exposure, whereas occupational health examination indicators, including blood pressure, metabolic markers, and anthropometric measurements, may reflect vulnerability or recovery-related strain. Linking these data sources with validated fatigue measures can operationalize an exposure–strain framework for workplace health surveillance in the future. Previous studies have examined the use of routine occupational health examinations and administrative data to strengthen surveillance capacity, although implementation research has identified limitations in the management of suspected occupational health risks [17,18]. Methodological guidance emphasizes that routine occupational health data should be collected and analyzed with clear objectives, standardized measurement procedures, quality control, and privacy protection [17,19]. These principles are particularly important when routine data are used to support decisions that affect workload planning and worker well-being.

Artificial intelligence and machine learning methods are increasingly being explored for occupational risk identification, health surveillance, and decision support [20,21,22,23]. However, predictive performance alone is insufficient for responsible workplace implementation of AI models. In occupational health settings, predictive tools must be interpretable, auditable, and embedded within human oversight to reduce the risk of misuse, functional creep, or punitive application. This requirement is especially important in contexts where overtime work may be financially valued by workers while also being associated with elevated fatigue and recovery-related risks. Therefore, a useful model should not replace the judgment of occupational health professionals. Rather, it should provide explainable probability estimates that support proportionate reviews, recovery planning, and preventive workload management.

Despite the growing interest in machine learning for occupational safety and health, few internally validated approaches have translated routinely collected working-hour trajectories and occupational health examination indicators into review priority tiers for preventive overtime review. Existing evidence has established associations between long working hours, fatigue, and adverse health outcomes; however, fewer studies have examined how routine organizational data can be converted into transparent screening signals that guide operational follow-up under human supervision. This gap is particularly relevant in high-intensity manufacturing workplaces, where overtime decisions are frequent, review capacity is limited, and timely identification of currently elevated work-related fatigue may support safer workload allocation.

To address this gap, this study developed and internally validated an explainable human-in-the-loop concurrent screening model for elevated work-related fatigue among manufacturing workers. The model integrates routinely collected working-hour records, occupational health examination indicators, demographic and occupational characteristics, and recovery-related variables to estimate the probability of elevated work-related fatigue at the index assessment. Multivariable logistic regression was compared with XGBoost using stratified cross-validation with out-of-fold estimated probability. Model performance was evaluated using discrimination, probabilistic accuracy, calibration, and screening metrics at pre-specified thresholds. Model interpretation was conducted using SHAP-based attribution, and model-estimated probabilities were mapped into low-, moderate-, and high-priority review tiers to support preventive overtime reviews under occupational health oversight. The intended use of the model is for concurrent screening and organizational prevention, rather than forecasting future fatigue onset, automated decision-making, clinical diagnosis, personnel evaluation, or punitive action.

## 2. Materials and Methods

### 2.1. Study Design, Setting, and Data Sources

This retrospective cross-sectional model development and internal validation study was conducted at a high-technology electronic equipment manufacturing company in northern Taiwan that employed approximately 350 workers during the study period. The study was designed to support prevention-oriented overtime reviews by developing an auditable, probability-based concurrent screening signal for identifying employees with currently elevated work-related fatigue at the index assessment. The model was not intended to predict future fatigue onset.

Eligible participants were full-time employees who completed both a routine annual occupational health examination and a standardized workplace fatigue assessment during the same two-year period. Specific calendar years are not reported to preserve the organizational confidentiality. A total of 326 employees were initially assessed for their eligibility. Of these, five were excluded because of incomplete fatigue assessments, three because of unavailable working-hour records or unsuccessful linkage across data sources, and four because of missing variables required for the primary analysis of complete cases. The final analytic sample comprised 314 employees, corresponding to a complete-case retention rate of 96.3%. A complete-case analysis was used, and no variable-level or item-level imputation was performed.

Employees were not excluded based on their work schedules. However, rotating and night-shift statuses were not separately retained in the de-identified analytic dataset and could therefore not be independently characterized. Occupational health examinations and workplace fatigue assessments were required to be completed within the same calendar month. Exact day-level intervals between the two assessments were not retained in the de-identified analytical dataset.

Three routinely collected data sources were linked for the analysis: occupational health examination records, administrative working-hour records, and standardized workplace fatigue questionnaire data. Occupational health examinations provided anthropometric measurements, blood pressure, lipid profiles, fasting glucose levels, and selected laboratory indicators of the participants. Administrative records provided the average daily working hours, monthly overtime hours, and internal overtime-threshold indicators.

The standardized workplace fatigue assessment was based on the Taiwanese Occupational Burnout Inventory, also referred to as the Workplace Fatigue Questionnaire, which was developed and validated for use among employed workers in Taiwan [24]. The company’s routine assessment included three five-item subscales measuring personal fatigue, work-related fatigue, and overcommitment. These three subscales were derived from the same validated instrument, and no items were modified or removed from the administered scale. Each item was rated on a five-point Likert-type response scale. Subscale scores were calculated as the mean of the five-item responses and linearly transformed to a 0–100 scale, with higher scores indicating greater fatigue or overcommitment. A subscale score was calculated only when all five items were answered. Employees with missing responses for any item required to define the work-related fatigue outcome or questionnaire-derived predictors were excluded from the complete-case analytic sample. Internal consistency was assessed separately for each administered subscale using Cronbach’s alpha. The final analytic sample included 78 employees with elevated work-related fatigue, corresponding to 24.8% of the analytic sample.

Data linkage was performed using pseudonymized employee identifiers. Authorized company personnel performed the linkage, and the linkage keys were stored separately by the company under restricted access. The research dataset provided to the investigators contained no direct or personal identifiers. All analyses were conducted on de-identified data within access-restricted systems, and the results were reported only in aggregate form to reduce the risk of re-identification.

### 2.2. Variables, Outcome Definition, and Model Specifications

Consistent with the TRIPOD+AI terminology, the present model was specified as a diagnostic or cross-sectional prediction model for concurrent screening rather than as a prognostic model. The outcome, high work-related fatigue, was ascertained at the index assessment concurrently with the occupational health examination indicators, whereas working-hour and overtime exposure variables were defined over the preceding one–seven months. Accordingly, the model estimates the probability of currently elevated work-related fatigue at the index assessment and is intended to support a timely review when overtime persists. However, it does not predict the future onset of fatigue. Prospective prognostic evaluation, in which fatigue outcomes are measured after the predictor window, should be prioritized in future research.

**Figure 1:**
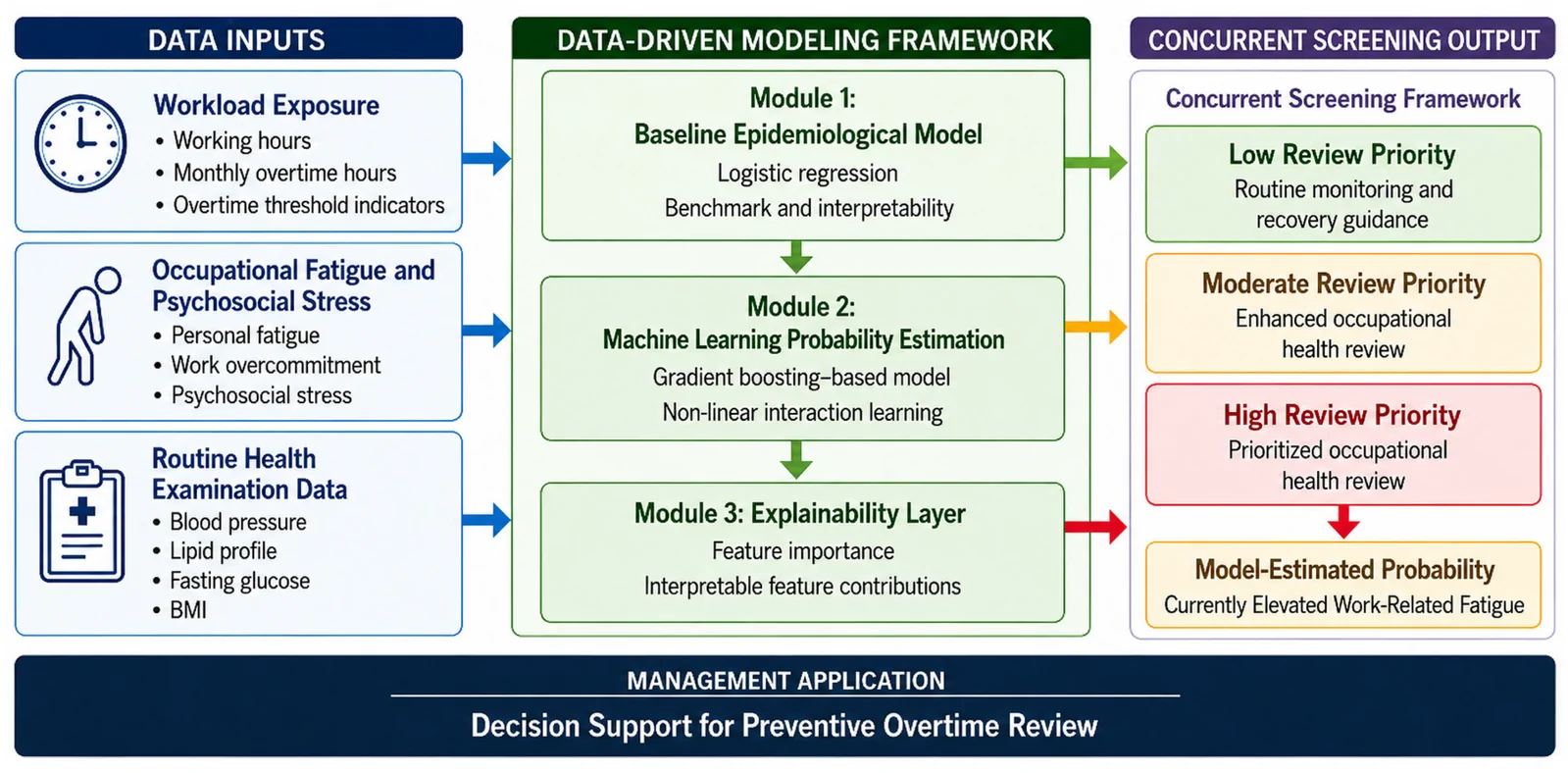
Conceptual framework and data integration pathway for explainable concurrent screening of currently elevated work-related fatigue and preventive overtime review. Routinely collected working-hour records, occupational health examination indicators, demographic and occupational characteristics, and recovery- and lifestyle-related variables were integrated to estimate the probability of currently elevated work-related fatigue at the index assessment. Model-estimated probabilities were mapped to provisional review-priority tiers to illustrate differentiated preventive overtime review under occupational health oversight and human decision-making.

Candidate predictors were prespecified based on a prevention-oriented cumulative exposure–strain framework linking overtime exposure, insufficient recovery, and vulnerability markers. The index assessment month was defined as the calendar month in which both the occupational health examination and the workplace fatigue questionnaire were completed, either on the same day or within the same month. All working-hour and overtime exposure variables were defined with respect to the index month. Figure 1 summarizes the conceptual framework and data integration pathway used to develop a concurrent screening model for currently elevated work-related fatigue.

For the primary routine data specification, 23 measured candidate variables were prespecified and represented by 25 model parameters after dummy coding of the four-level job-category variable. Candidate predictors were organized into four domains: workload and overtime exposure, demographic and occupational characteristics, recovery and lifestyle factors, and occupational health examination indicators.

The workload and overtime domains comprised seven parameters. The average daily working hours during the index assessment month were obtained from the company’s administrative attendance records and expressed continuously in hours per day. For descriptive reporting, the average daily working hours were grouped as <8, 8–10, and ≥10 h/day. Overtime exposure was also obtained from administrative working-hour records and included overtime hours during month −1, cumulative overtime hours during months −2 to −6, and cumulative overtime hours during months −1 to −7, all of which were expressed in hours. Three additional binary indicators identified whether an employee met or exceeded 37, 72, or 92 overtime hours in any month from month −1 to month −7. These indicators were coded as 0 = no and 1 = yes, with no threshold exceedance as the reference.

The demographic and occupational domains comprised age, sex, employment tenure, and job category. Age and employment tenure were expressed continuously in years and therefore had no reference category. Sex was coded as 0 = female and 1 = male, with females as the reference category. Job categories were classified as administration, sales, research and development, or manufacturing. Administration was used as the reference category, and sales, research and development, and manufacturing were represented by three separate binary indicator variables. Accordingly, the four measured demographic and occupational variables contributed to the six model parameters.

The recovery and lifestyle domains included sleep duration, weekly exercise amount, smoking status, and alcohol use. Sleep duration was obtained from a single self-reported item asking employees to report their usual daily sleep duration. It was expressed continuously in hours per day and, therefore, had no reference category. For descriptive reporting, sleep duration was grouped as <6, 6–8, and ≥8 h/day. The weekly exercise amount was obtained from a single self-report item asking employees to report their usual amount of exercise per week and was expressed in hours per week. For descriptive reporting, employees who reported any weekly exercise were summarized as engaging in regular exercise. Smoking status was coded as 0 = non-smoking and 1 = smoking, with non-smoking being the reference category. Alcohol use was coded as 0 = no alcohol use and 1 = alcohol use, with no alcohol use being the reference category.

The occupational health examination domain included eight continuously measured indicators: body mass index, systolic blood pressure, diastolic blood pressure, fasting glucose, triglyceride, total cholesterol, high-density lipoprotein cholesterol, and low-density lipoprotein cholesterol. These measurements were obtained from routine annual occupational health examinations corresponding to the index assessment. Body mass index is expressed in kg/m^2^, systolic and diastolic blood pressure in mmHg, and fasting glucose, triglycerides, total cholesterol, high-density lipoprotein cholesterol, and low-density lipoprotein cholesterol in mg/dL. These indicators had no reference categories and were interpreted as routinely measured markers of vulnerability or recovery-related strain for screening rather than diagnostic endpoints.

Accordingly, the 25 candidate predictor parameters in the primary routine-data specification were average daily working hours; overtime hours during month −1; cumulative overtime hours during months −2 to −6; cumulative overtime hours during months −1 to −7; indicators for meeting or exceeding 37, 72, and 92 overtime hours per month; age; sex; employment tenure; sales, research and development, and manufacturing job-category indicators; sleep duration; weekly exercise amount; smoking status; alcohol use; body mass index; systolic blood pressure; diastolic blood pressure; fasting glucose; triglycerides; total cholesterol; high-density lipoprotein cholesterol; and low-density lipoprotein cholesterol. Outlier screening, variable encoding, and standardization procedures are described in Section 2.3.

The primary outcome was elevated work-related fatigue at the index assessment. This outcome was defined a priori using the top quartile of the standardized work-related fatigue subscale, representing a screening-oriented outcome definition rather than a clinical diagnosis of WRF. In the analytic sample, the empirical 75th-percentile cutoff corresponded to a work-related fatigue score of 46.0 on a 0–100 scale. Employees with scores above this cutoff were classified as having elevated work-related fatigue. No employee had a score exactly equal to 46.0; therefore, no ties occurred at the cut-off. Using this definition, 78 of the 314 employees (24.8%) were identified as having elevated work-related fatigue. A percentile-based definition was selected to identify a manageable subgroup requiring an enhanced preventive review under occupational health capacity constraints. To reduce information leakage, the work-related fatigue score used to define the outcome was excluded from the predictor sets.

Two prespecified model specifications were evaluated. The primary routine data specification included the 25 candidate predictor parameters described above and was intended to represent a feasible screening scenario based on routinely available administrative, occupational, lifestyle, and occupational health data. The secondary questionnaire-enhanced specification included all predictors from the primary specification and additionally incorporated personal fatigue and work overcommitment as continuous 0–100 predictors, resulting in 27 candidate parameters. The work-related fatigue score used to define the outcome was excluded from both the specifications. The secondary specification was used to evaluate the incremental contribution of questionnaire-derived psychosocial measures and to support mechanistic interpretation while recognizing the potential for reporting bias and common method concerns.

### 2.3. Model Development and Internal Validation

Multivariable logistic regression was compared with gradient-boosted decision trees implemented using XGBoost. Primary inferences were based on routine data specifications. The logistic regression model included all prespecified candidate model parameters without univariable screening, stepwise selection, or other data-driven variable selection procedures. This was a deliberate methodological decision. Given the limited sample size and information density, data-driven screening or stepwise selection could produce unstable predictor sets and additional selection-induced overfitting or optimism. Prespecifying the candidate set preserved the cumulative exposure–strain framework and facilitated consistent interpretation across modeling approaches, although this choice reduced model parsimony and may have retained weakly informative or partially redundant predictors in the model. Therefore, the resulting models should not be interpreted as identifying a minimal set of predictors. The logistic regression benchmark was fitted with L2 penalization using the L-BFGS solver and an inverse regularization strength of *C* = 1.0. The regularization strength was fixed and not tuned within the cross-validation procedure.

For the logistic regression, continuous predictors were standardized to z-scores using the mean and standard deviation estimated exclusively from the relevant training partition. The resulting training-derived standardization parameters were then applied to the corresponding validation or held out partition. Binary variables and dummy-coded job category indicators were not standardized. For XGBoost, continuous predictors were retained in their original measurement units because tree-based models do not require the standardization of features. The same binary coding and job-category reference coding described in Section 2.2 were used for both the modeling approaches.

Before cross-validation, continuous variables were screened using prespecified, non-data-adaptive checks for administratively impossible or physiologically implausible values of the variables. Invalid values were treated as missing and handled according to the complete-case rule. Valid observations were retained even when they were statistically extreme. No statistical outlier trimming, winsorization, or percentile-based exclusion was performed.

Potential predictor redundancy and multicollinearity were assessed descriptively to characterize the correlation structure of the prespecified set of predictors. Pairwise Spearman correlation coefficients were calculated among continuous predictors, with particular attention to the structurally overlapping cumulative overtime variables for months −2 to −6 and −1 to −7 and to systolic and diastolic blood pressure. Multicollinearity in the logistic regression design matrix was assessed using variance inflation factors (VIFs) to evaluate whether correlated predictors could materially affect the stability and interpretation of individual regression coefficients. These diagnostics were used only to inform the interpretation of correlated predictors and were not used for feature elimination, model specification, or hyperparameter tuning. Accordingly, the prespecified predictor set was retained in both modeling approaches.

All fold-dependent preprocessing procedures were nested within the corresponding resampling framework. During the outer cross-validation, the preprocessing parameters and class weights were estimated without access to the outer held-out fold. During inner cross-validation for XGBoost hyperparameter tuning, any fold-dependent preprocessing parameters and class weights were estimated using only the corresponding inner training fold and were subsequently applied to the inner validation folds. After hyperparameter selection, the preprocessing parameters and class weights were re-estimated using the complete outer training partition before evaluation in the outer held-out fold.

The analytic sample comprised 314 employees, including 78 employees with currently elevated work-related fatigue, corresponding to an event fraction of 0.248. The routine data specification included 25 candidate model parameters, yielding approximately 3.12 events per parameter. This modest event-to-parameter ratio indicates limited information density, which may reduce the stability of fitted model relationships and increase the risk of optimism. Because a fixed events-per-parameter rule alone is insufficient for determining prediction-model sample size adequacy, the minimum required sample size was additionally evaluated according to the criteria proposed by Riley et al. [25], based on the anticipated Cox–Snell *R*^2^ and a plausible discrimination range corresponding to a C-statistic of approximately 0.68–0.77.

The available sample satisfied the criterion for the precise estimation of the overall outcome proportion, for which the estimated required sample size was approximately 287. However, it did not satisfy the more stringent criterion of maintaining a global shrinkage above 0.90 and limiting optimism in the model fit. Depending on the assumed number of parameters and anticipated discrimination, the estimated required sample size was approximately 1000–3400 participants. Accordingly, the models were interpreted as exploratory, internally validated screening models developed within a single organization, rather than as definitive or implementation-ready prediction tools. Logistic regression was retained as a transparent benchmark, predictors were prespecified without data-driven selection, and XGBoost complexity was controlled through nested tuning of the number of boosting trees, learning rate, tree depth, minimum child weight, gamma, row and feature subsampling, and L1/L2 leaf weight penalties.

Internal validation used nested cross-validation, comprising stratified five-fold outer cross-validation for performance estimation and stratified five-fold inner cross-validation for XGBoost hyperparameter selection. Within each outer fold, all fold-dependent preprocessing procedures, model fitting, class weight calculation, and hyperparameter tuning were restricted to the outer training partition. Predictions were subsequently generated for the corresponding outer-held-out fold. Aggregated out-of-fold predictions from the five outer folds were used for all reported discrimination, calibration, and threshold-dependent performance estimates to reduce the optimism and minimize information leakage.

For XGBoost, the hyperparameters were tuned separately within each outer training partition using a random search with 150 candidate configurations and stratified five-fold inner cross-validation. The prespecified search space included n_estimators = 100, 150, 200, 250, 300, or 400; learning_rate = 0.01, 0.03, 0.05, 0.08, or 0.10; max_depth = 2, 3, 4, or 5; min_child_weight = 1, 3, 5, 7, or 10; subsample = 0.60, 0.70, 0.80, 0.90, or 1.00; colsample_bytree = 0.60, 0.70, 0.80, 0.90, or 1.00; gamma = 0, 0.10, 0.25, 0.50, or 1.00; reg_alpha = 0, 0.01, 0.10, 0.50, or 1.00; and reg_lambda = 0.50, 1.00, 2.00, 5.00, or 10.00. The mean inner-fold area under the receiver operating characteristic curve was used as the hyperparameter-selection criterion. Across the five outer folds, the selected hyperparameters had the following median (range): n_estimators, 250 (200–300); learning_rate, 0.05 (0.03–0.08); max_depth, 3 (2–4); min_child_weight, 5 (3–7); subsample, 0.80 (0.70–0.90); colsample_bytree, 0.80 (0.70–0.90); gamma, 0.25 (0.10–0.50); reg_alpha, 0.10 (0–0.50); and reg_lambda, 2.00 (1.00–5.00). For each candidate configuration, model fitting and fold-dependent preprocessing were performed using only the corresponding inner training fold, and the performance was evaluated in the inner validation fold. The configuration with the highest mean inner fold AUC was then refitted using the complete outer training partition before predictions were generated for the corresponding outer held-out fold.

Here, “within-fold regularization” refers to the XGBoost-native complexity-control and regularization mechanisms that were tuned exclusively within the inner cross-validation procedure of each outer training partition. These included the control of the number of boosting trees and boosting step size, constraints on the maximum tree depth and minimum child weight, minimum loss reduction required for additional splitting (gamma), row and feature subsampling (subsample and colsample_bytree), and L1 and L2 penalties on leaf weights (reg_alpha and reg_lambda, respectively). All hyperparameter selections were based exclusively on the mean inner-fold AUC without access to the corresponding outer held-out fold. No separate post hoc regularization, shrinkage, pruning, or coefficient adjustment procedures were applied.

Class weights were applied during model fitting to address the class imbalance associated with the 24.8% event rate. Within the nested validation framework, class weights were determined using only the relevant training data and were not estimated using outcomes from either the inner validation folds or the outer held-out fold.

Two study-specific thresholds, τ1 = 0.28 and τ2 = 0.55, were used to construct provisional low-, moderate-, and high-priority tiers. Threshold selection and evaluation were conducted using the same development dataset and its aggregated out-of-fold predictions. Consequently, threshold-based classification metrics, tier sizes, and observed fatigue prevalence within tiers should be interpreted as exploratory internal estimates rather than independent evidence of threshold stability and transportability. The thresholds were not externally validated and were not intended to serve as fixed decision boundaries for direct, operational use.

Reporting was aligned with the TRIPOD+AI recommendations for prediction model development and internal validation using regression and machine learning methods [26].

### 2.4. Model Performance, Calibration, and Interpretability

Model discrimination was assessed using the area under the receiver-operating characteristic curve. The overall probabilistic accuracy was evaluated using the Brier score. The classification performance was summarized using sensitivity, specificity, precision, and F1 score at a study-specific threshold of τ1 = 0.28. This threshold was chosen to prioritize sensitivity for concurrent identification in a screening context while maintaining a feasible review burden for occupational health services. Uncertainty in key performance metrics was quantified at the employee level using nonparametric bootstrapping with 2000 resamples to obtain 95% confidence intervals. Because the two models were evaluated using the same aggregated out-of-fold predictions, the difference in AUC was assessed as a paired comparison using the DeLong test [27], with a paired bootstrap resampling of 2000 resamples for confirmatory analysis. The overlap between the two marginal confidence intervals was not used to determine the difference, as this approach does not account for the correlation between paired estimates.

The calibration was evaluated using aggregated out-of-fold predictions. The calibration-in-the-large and calibration slope were estimated using a logistic recalibration model, in which the currently elevated work-related fatigue status was regressed on the logit of the model-estimated probability. Calibration plots were generated by grouping the predicted probabilities into quantiles and comparing the mean model-estimated probability with the observed event rate in each group. Bootstrap resampling with 2000 resamples was used to estimate the uncertainty of the calibration parameters. Because the intended application of the model involves probability thresholds and provisional review-priority tiering, calibration was considered essential for evaluating potential future implementation.

The interpretability of the model was examined using SHAP values. To reduce the risk of information leakage, the SHAP values were computed separately for each held-out fold using the corresponding fold-specific XGBoost model. The fold-specific SHAP values were then aggregated across the folds to summarize the relative contribution of the predictors to the model output. The interpretation focused on whether the most influential predictors were consistent with the cumulative exposure–strain framework and appropriate for occupational health review under human oversight. All analyses were conducted using Python version 3.11.13 with scikit-learn version 1.6.1, XGBoost version 2.1.4, SHAP version 0.47.2, NumPy version 2.2.6, pandas version 2.3.0, and SciPy version 1.15.3.

### 2.5. Provisional Review-Priority Stratification

For provisional review-priority stratification, the XGBoost model-estimated probabilities of currently elevated work-related fatigue were grouped using two study-specific thresholds, τ1 = 0.28 and τ2 = 0.55. Employees with model-estimated probabilities below τ1 were assigned to the low-priority tier; those with probabilities from τ1 to below τ2 were assigned to the moderate-priority tier; and those with probabilities greater than or equal to τ2 were assigned to the high-priority tier. The lower threshold was selected to prioritize sensitivity for concurrent screening, whereas the higher threshold was intended to identify a smaller subgroup for a prioritized occupational health review.

These provisional tiers were used to illustrate how different levels of occupational health review might be allocated, including routine monitoring, enhanced review, recovery support planning, and occupational health-led follow-up. They were not intended to serve as diagnostic labels, automated employment decisions, fitness-for-work determinations, or fixed criteria for personnel evaluation or for overtime eligibility.

### 2.6. Ethics and Data Protection

This study used secondary data originally collected for routine occupational health management by the participating company. No participant contact was involved, and all direct identifiers were removed before the investigators accessed the analytical dataset. Authorized company personnel performed data linkage using pseudonymized employee identifiers, and the linkage keys were stored separately by the company under restricted access. The research dataset provided to the investigators contained no direct or personal identifiers, and the investigators had no access to the linkage keys or means of reidentifying individual workers. All analyses were conducted within access-restricted systems, and the results were reported only in aggregate form.

Formal institutional review board approval was not obtained for this retrospective secondary analysis of de-identified occupational health data. The study protocol, secondary-use purpose, and data handling procedures were reviewed and authorized by the participating company’s Occupational Safety and Health Committee under the company’s occupational health management and data governance procedures. This organizational authorization constituted the company’s internal governance pathway for the secondary use of de-identified occupational health data and did not constitute an independent institutional review board review or a formal IRB exemption determination. No prospective intervention, automated employment decision, or employee-level action was undertaken as part of the present study.

The model outputs are intended only for concurrent occupational health screening and decision support under human oversight. They are not intended for automated decision-making, clinical diagnosis, personnel evaluation, remuneration decisions, disciplinary actions, or punitive use.

## 3. Results

### 3.1. Study Population and Descriptive Characteristics

Of the 326 employees initially assessed for eligibility, five were excluded because of incomplete fatigue assessments, three because of unavailable working-hour records or unsuccessful linkage across data sources, and four because of missing variables required for the primary analysis of complete cases. The final analytic sample comprised 314 employees, corresponding to a complete-case retention rate of 96.3%.

Among the 318 employees with successfully linked questionnaires, working hours, and occupational health records, variable-level missingness ranged from 0% to 1.2%. No missing data were identified for age, sex, employment tenure, job category, systolic blood pressure, diastolic blood pressure, or work-related fatigue outcomes. Missingness was 0.3% for average daily working hours, weekly exercise amount, and body mass index; 0.6% for monthly overtime records, sleep duration, smoking status, and alcohol use; and ranged from 0.3% to 1.2% across the selected laboratory indicators. As missing values overlapped across variables, four employees were excluded from the complete-case analysis. Descriptive comparisons based on available age, sex, employment tenure, job category, and average daily working-hour data indicated no material differences between the included and excluded employees; however, the small number of excluded employees limited the assessment of potential selection bias.

Among the 314 employees included, 78 (24.8%) met the criterion for currently elevated work-related fatigue, whereas 236 (75.2%) were classified as not having currently elevated fatigue. Participants were predominantly male (65.3%), and 40.8% had an employment tenure of at least ten years. The largest job categories were manufacturing (37.6%) and R&D (37.3%). Most employees worked an average of 8–10 h per day (79.6%); 15.0% worked fewer than 8 h per day, and 5.4% worked 10 h or more per day. Regular exercise was reported by 77.4% of the employees, and 94.6% reported sleeping 6–8 h per day.

### 3.2. Fatigue and Overcommitment by Sex and Working-Hour Category

The three questionnaire subscales demonstrated good to excellent internal consistency in the present analytic sample. Cronbach’s alpha values were 0.87, 0.92, and 0.88 for personal fatigue, work-related fatigue, and work overcommitment, respectively. Table 1 shows the fatigue and overcommitment scores stratified by sex and average daily working-hour category. Personal and work-related fatigue did not differ significantly between men and women (*p* = 0.104 and *p* = 0.209, respectively). However, work overcommitment was significantly higher among men than among women (42.9 vs. 37.9; *p* = 0.020). Across average daily working-hour categories, work overcommitment also differed significantly (*p* = 0.034), with the highest scores observed among employees working ≥ 10 h per day. In contrast, personal and work-related fatigue did not differ significantly across working-hour categories.

### 3.3. Overtime Exposure and Internal Monitoring Thresholds

Table 2 summarizes the overtime exposure indicators and the frequency of exceeding internal overtime monitoring thresholds by fatigue group. Employees in the high-fatigue group had a higher median overtime across all prespecified exposure windows. In month −1, the median monthly overtime was 33 h (interquartile range [IQR], 18–55 h) among employees with high fatigue, compared with 22 h (IQR, 10–38 h) among those without high fatigue. For cumulative overtime during months −2 to −6, the corresponding medians were 170 h (IQR, 105–245 h) and 112 h (IQR, 60–175 h). Across the extended exposure window from months −1 to −7, employees in the high-fatigue group had a median cumulative overtime of 235 h (IQR, 140–320 h), compared with 160 h (IQR, 85–240 h) among employees without high fatigue levels.

Exceedance of internal overtime monitoring thresholds was also more frequent in the high fatigue group. The ≥37 overtime hours/month threshold was exceeded by 39.7% of employees with high fatigue compared with 25.8% of those without high fatigue. Similar directional patterns were observed for the ≥72 and ≥92 h/month thresholds, which were exceeded by 12.8% and 6.4% of employees with high fatigue, respectively, compared with 5.9% and 2.1% of employees without high fatigue, respectively.

### 3.4. Internal Validation Performance of the Routine-Data Specification

**Figure 2:**
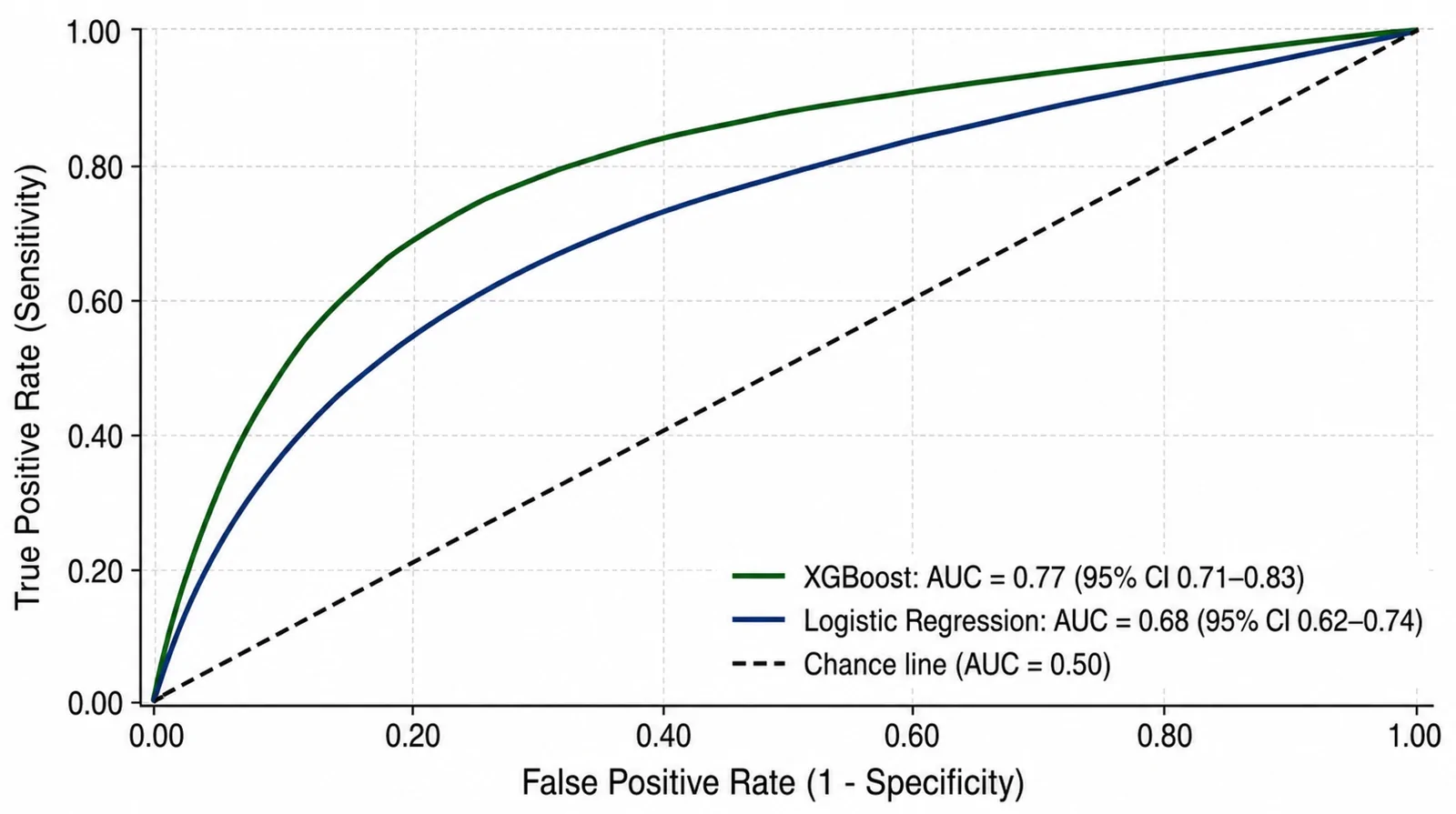
Receiver operating characteristic curves for the internal validation of routine data specifications.

Table 3 and Figure 2 summarize the internal validation results for the routine data specifications based on stratified five-fold cross-validation with aggregated out-of-fold predictions. XGBoost achieved higher discrimination than logistic regression, with an AUC of 0.77 (95% CI, 0.71–0.83) compared to 0.68 (95% CI, 0.62–0.74). Because both models were evaluated using the same out-of-fold predictions, the difference in AUC was assessed using a paired comparison rather than by examining the overlap between the marginal confidence intervals. The paired AUC difference was 0.09, indicating significantly higher discrimination for XGBoost than for logistic regression, according to the DeLong test (*p* < 0.01).

At the study-specific provisional threshold of τ1 = 0.28, XGBoost achieved a sensitivity of 0.78, specificity of 0.70, accuracy of 0.72, precision of 0.46 and F1 score of 0.58. In comparison, logistic regression achieved a sensitivity of 0.67, specificity of 0.69, accuracy of 0.68, precision of 0.42, and F1 score of 0.51. These results suggest that the XGBoost model offers improved discrimination, overall probabilistic accuracy, and sensitivity for prevention-oriented screening while maintaining comparable specificity. Because τ1 was selected and evaluated using the same dataset, these threshold-dependent performance estimates may be optimistic and require confirmation in independent prospective and external samples.

### 3.5. Calibration Assessment

**Figure 3:**
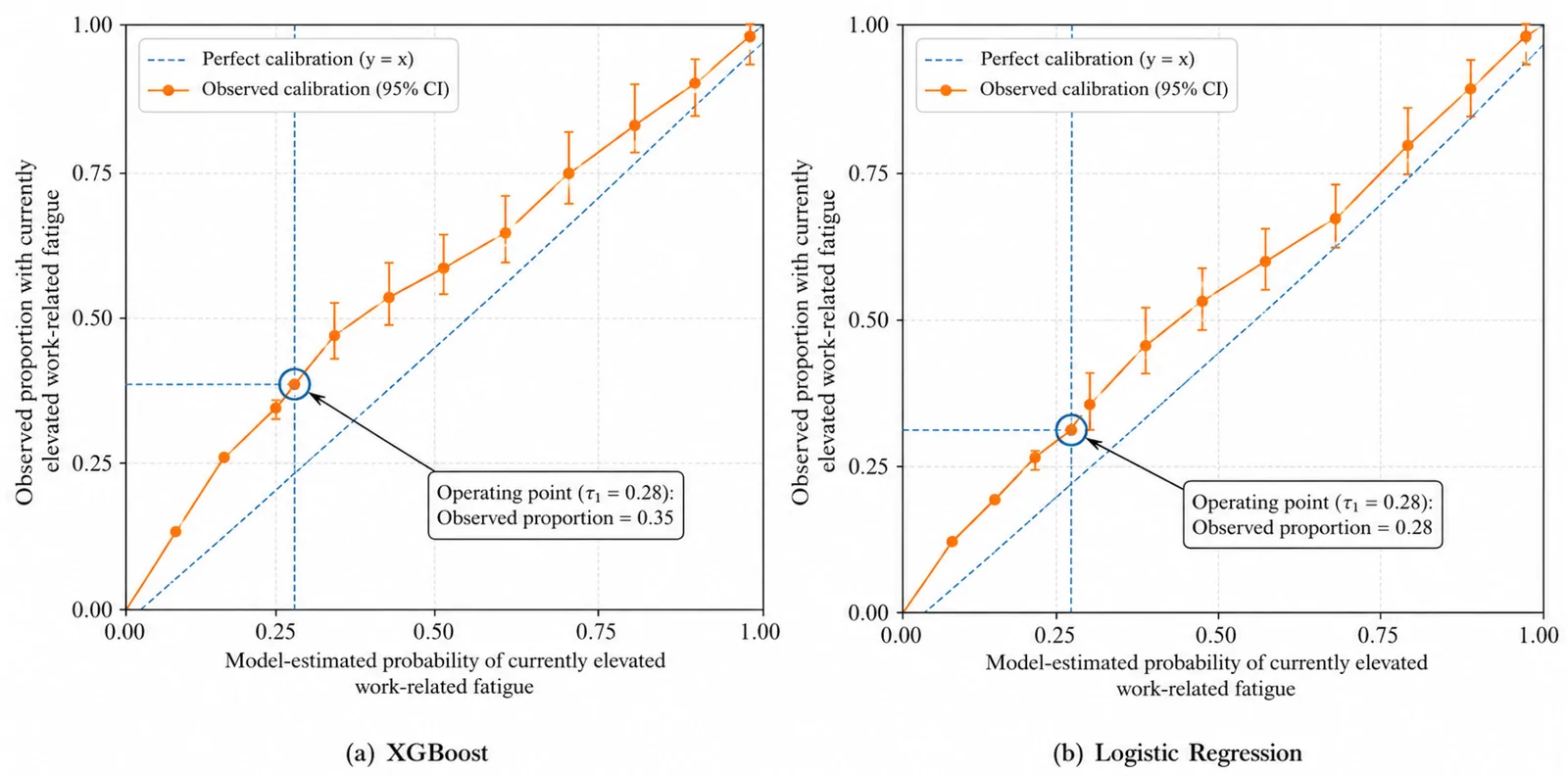
Calibration plots of the routine data specification using aggregated out-of-fold predictions.

Because the intended application of the model is preventive screening and triage rather than clinical diagnosis, calibration was assessed using aggregated out-of-fold (OOF) predictions. Both models showed evidence of systematic miscalibration (Figure 3). XGBoost had a calibration intercept of α = 0.78 (95% CI, 0.24–1.35) and a calibration slope of β = 1.64 (95% CI, 1.13–2.24). In contrast, the logistic regression had a calibration intercept of α = −0.49 (95% CI, −0.84 to −0.15) and a calibration slope of β = 0.56 (95% CI, 0.32–0.81).

These findings indicate that, although XGBoost achieved better discrimination and probabilistic accuracy, its probability estimates should be recalibrated before a threshold-based operational implementation. Accordingly, the application of probability thresholds for governance should include intercept and slope recalibration, along with ongoing monitoring of calibration performance and review-priority tier distributions.

### 3.6. Model Interpretability and Secondary Questionnaire-Enhanced Analysis

Correlation and collinearity diagnostics revealed the expected structural redundancy among several related predictors. Cumulative overtime during months −2 to −6 and −1 to −7 was strongly correlated (Spearman *ρ* = 0.85), consistent with the substantial overlap between these exposure windows. Systolic and diastolic blood pressures were also positively correlated (*ρ* = 0.70). The corresponding variance inflation factors (VIFs) were 4.6 and 4.8 for cumulative overtime during months −2 to −6 and months −1 to −7, respectively, and 2.1 and 2.0 for systolic and diastolic blood pressure, respectively. The maximum VIF among the prespecified predictors was 4.8. Although these diagnostics did not indicate severe multicollinearity, they confirmed that several related predictors contained overlapping information, and therefore warranted cautious interpretation of individual feature contributions.

**Figure 4:**
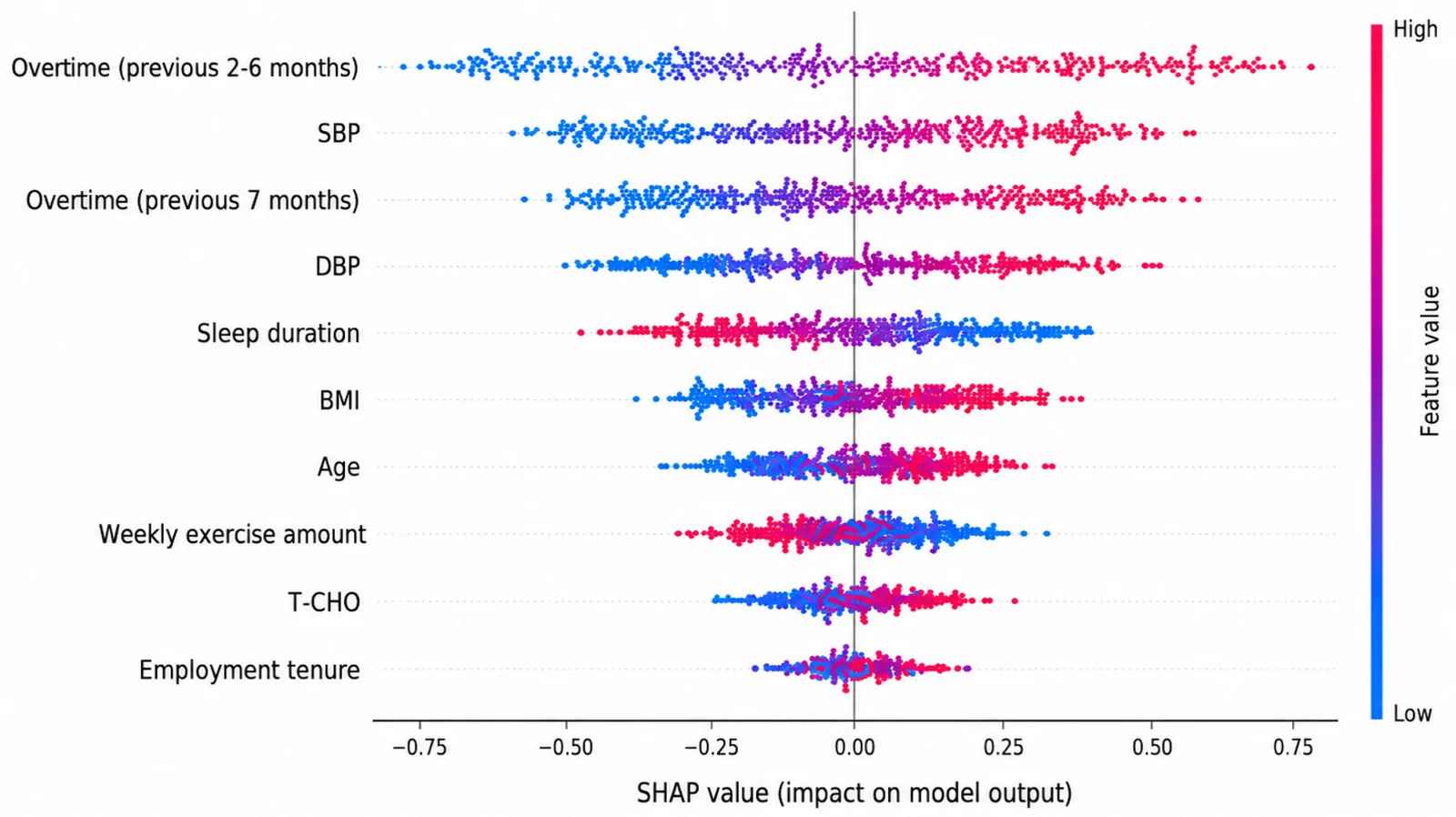
SHAP summary plot for the XGBoost model estimating the probability of currently elevated work-related fatigue.

Figure 4 shows the SHAP summary plot for the XGBoost routine data model. The plot shows the ten most influential features ranked by the mean absolute SHAP value. Each point represents an individual employee, and the horizontal axis indicates the direction and magnitude of the feature’s contribution to the model-estimated probability of elevated work-related fatigue. Points positioned to the right indicate an increase in the model-estimated probability, whereas points positioned to the left indicate a decrease in the model-estimated probability. The point color denotes the observed feature value, with lower values shown in blue and higher values shown in red.

The five most influential features were cumulative overtime during months −2 to −6 (mean |SHAP| = 0.085), systolic blood pressure (0.070), cumulative overtime during months −1 to −7 (0.050), diastolic blood pressure (0.040), and sleep duration (0.030). Other influential predictors included body mass index, age, weekly exercise amount, total cholesterol, and employment tenure. Although cumulative overtime during months −2 to −6 had a larger mean absolute SHAP attribution than the broader months −1 to −7 exposure window, the two variables substantially overlapped and were strongly correlated. Therefore, their individual SHAP rankings should not be interpreted as independent measures of relative importance. Rather, their combined prominence supports the interpretation that cumulative overtime exposure is an influential feature domain in the fitted model. Similarly, because systolic and diastolic blood pressure are correlated, their individual SHAP attributions may partly reflect shared or redistributed information across related physiological features.

The SHAP patterns for the cumulative overtime variables generally indicated that higher cumulative overtime exposure contributed to a higher model-estimated fatigue probability, whereas lower cumulative exposure tended to contribute in the opposite direction. Given the strong correlation between the two cumulative exposure windows, these patterns are most appropriately interpreted jointly as reflecting the contribution of cumulative overtime exposure rather than as the independent effects of the individual windows. Systolic and diastolic blood pressure also contributed to the model output; given their correlation, these measures are best interpreted jointly as representing a blood pressure-related physiological domain rather than as independent physiological effects. Sleep duration contributed in the expected direction, with longer sleep duration generally associated with a lower model-estimated fatigue probability.

In the secondary questionnaire-enhanced specification, overcommitment added interpretive value, consistent with a psychosocial vulnerability pathway; however, questionnaire-derived predictors were excluded from the primary routine data specification. This incremental value should be interpreted with caution. Personal and work-related fatigue were derived from the same self-report questionnaire administered at a single time point; therefore, their association may partly reflect shared methods and instrument variance rather than independent predictive information. Therefore, questionnaire-derived predictors were excluded from the primary routine data specification, and the questionnaire-enhanced results are reported only to support mechanistic interpretation.

Each point represents an individual employee’s data. The point color denotes the observed feature value, with low values in blue and high values in red. The *x*-axis indicates each feature’s contribution to the model-estimated probability of currently elevated work-related fatigue. The features were ranked according to the mean absolute SHAP value. SBP, systolic blood pressure; DBP, diastolic blood pressure; BMI, body mass index; T-CHO, total cholesterol.

### 3.7. Provisional Review-Priority Tiering for Preventive Overtime Review

Using prespecified thresholds of τ1 = 0.28 and τ2 = 0.55, the XGBoost-estimated probabilities of currently elevated work-related fatigue from the routine data model were assigned to three provisional review-priority tiers for preventive overtime reviews (Table 4). Overall, 182 employees (58.0%) were classified into the low-priority tier, 85 (27.1%) into the moderate-priority tier, and 47 (15.0%) into the high-priority tier. The high-priority tier comprised approximately 15% of employees, consistent with the prespecified review-capacity target. This finding illustrates a potential capacity-sensitive prioritization framework but does not establish optimal or effective alignment with actual occupational health service capacity.

The observed prevalence of elevated work-related fatigue increased across the three review-priority tiers. The low-priority tier had an observed prevalence of 9.3%, compared with 28.2% in the moderate-priority tier. The highest observed prevalence was found in the high-priority tier (78.7%). This gradient demonstrates internal differentiation across provisional review-priority tiers but does not establish external validity or implementation readiness.

When τ1 = 0.28 was applied as the screening threshold, employees in the moderate- and high-priority tiers were classified as screen positive. This classification produced 61 true positives, 71 false positives, 165 true negatives, and 17 false negatives, corresponding to a sensitivity of 0.78, specificity of 0.70, precision of 0.46, and F1 score of 0.58. These values are consistent with the XGBoost performance metrics listed in Table 3.

## 4. Discussion

This study developed and internally validated an explainable, human-in-the-loop concurrent screening model using routinely collected working-hour records and occupational health examination data to identify employees with elevated work-related fatigue. Its main contribution is the translation of routinely available workplace and occupational health data into explainable probability-based screening estimates and provisional review-priority tiers that may inform proportionate occupational health reviews following appropriate validation and recalibration. Rather than serving as a diagnostic tool, automated decision system, or prognostic model, the proposed model is intended to provide an auditable concurrent screening signal for workload governance, recovery planning, and preventive follow-up. It estimates the probability of elevated fatigue at the index assessment and should not be interpreted as a forecast of future fatigue onset. This orientation is consistent with the broader occupational health and fatigue management literature, which emphasizes prevention, ongoing monitoring, and organizational controls rather than reactive case management alone [16,28].

The present model should be interpreted as a cross-sectional screening tool, rather than a prognostic prediction model. Although several working-hour variables summarized overtime exposure during the months preceding the index assessment, the fatigue outcome was measured at the same index assessment as the occupational health examination indicators. Therefore, the model estimates the probability that an employee has elevated work-related fatigue at the time of assessment. However, it does not estimate the probability of future fatigue onset. Prospective studies with fatigue outcomes measured after the predictor window are required before prognostic claims can be made.

Several findings are noteworthy in this study. First, employees classified as having high work-related fatigue had greater overtime exposure across all prespecified windows, particularly for medium-term cumulative overtime during months −2 to −6 and extended cumulative overtime during months −1 to −7 (Table 2). This pattern supports the cumulative exposure–strain framework of this study. Long working hours have been consistently associated with adverse health outcomes, including cardiovascular morbidity and mortality, across diverse working populations [3,4,5,6]. The WHO–ILO joint estimates further indicate that exposure to working 55 or more hours per week contributes substantially to the global burden of ischemic heart disease and stroke [6]. In this context, repeated overtime exposure over several months may be more informative than a single-month measure, as it better reflects the accumulated recovery deficits and sustained job demands.

Second, work overcommitment showed a clearer association with the working-hour category than personal fatigue or work-related fatigue scores (Table 1). Overcommitment was highest among employees working ≥ 10 h per day, whereas cross-sectional personal and work-related fatigue scores showed less variation across working-hour groups. This finding is consistent with occupational health psychology perspectives suggesting that psychosocial strain, sustained effort investment, and insufficient recovery may emerge before overt fatigue is consistently captured at a single assessment point [8,11]. This also supports the value of integrating exposure trajectories with occupational health indicators rather than relying solely on one-time self-reported fatigue measures. Reviews of occupational fatigue assessment have noted that fatigue is multidimensional and context-dependent and that single instruments may have limited transferability across settings [12,15].

Third, the machine learning model improved the screening performance compared with the traditional logistic regression baseline. In the internal validation using stratified five-fold cross-validation and aggregated out-of-fold predictions, XGBoost achieved higher discrimination and better probabilistic accuracy than logistic regression (Table 3; Figure 2). At the study-specific provisional threshold of τ1 = 0.28, XGBoost achieved higher sensitivity while maintaining a comparable specificity. This trade-off may be useful for prevention-oriented screening, where missed currently elevated fatigue cases could delay occupational health review and recovery-support actions. However, consistent with the guidance on reporting predictive models, discrimination alone is insufficient to determine whether a model is suitable for implementation; calibration, transparency, and intended-use boundaries must also be considered [26,29].

Model interpretability also helps clarify why recovery-related and physiological variables may provide information beyond working-hour records alone. SHAP analysis showed that cumulative overtime exposure was a major contributor to the model output, with the − 2 to − 6 months window showing the largest mean absolute SHAP attribution (Figure 4). However, because the months −2 to −6 and −1 to −7 cumulative overtime variables overlap substantially by construction, their individual SHAP rankings should not be interpreted as independent measures of relative importance. Rather, the two variables jointly support the interpretation that cumulative overtime exposure constitutes an influential feature domain in the model. Administrative working-hour records quantify the external dose and duration of workload exposure; however, they do not directly capture recovery opportunities or individual responses to the same workload. Therefore, sleep duration may provide complementary information by reflecting recovery opportunities, sleep restriction, and circadian disruption that cannot be inferred from overtime totals alone. Evidence across occupational populations has similarly shown close interrelationships among work schedules, sleep, and fatigue, supporting the interpretation of exposure and recovery as related but distinct dimensions of occupational fatigue risk [8,9,30].

The amount of weekly exercise and routine physiological indicators may represent additional complementary dimensions. Exercise behavior may reflect physical conditioning, recovery behavior, and an employee’s capacity to recover from sustained work demands, rather than workload exposure itself. Experimental evidence in employees with work-related fatigue suggests that structured exercise can improve fatigue-related outcomes and sleep, although exercise should not be interpreted as a causal protective factor from the present cross-sectional data [31]. Routine occupational health indicators provide different types of information. Systolic and diastolic blood pressure were among the most influential features in the present model, whereas body mass index and total cholesterol contributed more modestly to the model. Because systolic and diastolic blood pressure are correlated physiological measures, their individual SHAP magnitudes may partly reflect shared or redistributed attributions across related features. Therefore, they should be interpreted jointly as indicators of a blood pressure-related physiological domain rather than as independent evidence of unique contributions to fatigue risk. More broadly, these routine physiological measures should be interpreted as nonspecific indicators of physiological state or cardiometabolic vulnerability, rather than fatigue-specific biomarkers or diagnostic endpoints. Their contribution suggests that employees with similar working-hour exposure may differ in underlying susceptibility or recovery-related strain.

The observed predictor correlations also have implications for model interpretation beyond the SHAP attribution. For correlated predictors, model attribution may be distributed across variables carrying overlapping information, and mean absolute SHAP values should therefore not be interpreted as independent effect sizes or causal effects. Similarly, multicollinearity can increase uncertainty and reduce the stability of the individual logistic regression coefficients. Although the use of L2 penalization may reduce coefficient instability, it does not eliminate the interpretive consequences of the correlated predictors. Accordingly, the logistic regression model was used primarily as a modeling benchmark, and the individual coefficients should not be interpreted as independent causal effects. Therefore, correlation and VIF diagnostics were used to qualify the interpretation rather than to support post hoc feature removal, consistent with the prespecified feature-selection strategy.

Routine hematological measures may represent another low-cost source of complementary physiological information for future models. For example, occupational research has reported associations between fatigue-related stress responses and white blood cell and neutrophil counts [32], whereas blood cell-derived indices, such as the neutrophil-to-lymphocyte ratio, have been used more broadly as readily available markers of systemic stress and inflammation [33]. These hematological indicators were not included in the present model, and their incremental predictive value for occupational fatigue screening should therefore be evaluated prospectively rather than inferred from our findings.

The calibration findings further clarify the model validation and implementation requirements. Although XGBoost outperformed logistic regression in terms of discrimination and Brier score, the calibration assessment showed systematic miscalibration (Figure 3). XGBoost had a calibration intercept of α = 0.78 and a calibration slope of β = 1.64, whereas logistic regression had α = −0.49 and β = 0.56. Calibration is particularly important when predicted probabilities are used to define decision thresholds because miscalibration may lead to inappropriate review burden, missed cases, or unnecessary intervention. Methodological literature on prediction models emphasizes that calibration-in-the-large, calibration slope, and calibration plots provide complementary information on whether the predicted risks align with the observed event rates [34]. Therefore, these findings caution against interpreting the current predicted probabilities or tier cut-off points as calibrated decision boundaries.

The provisional tiering approach illustrates how predicted probabilities might eventually support differentiated levels of occupational health reviews. The observed prevalence of currently elevated work-related fatigue increased across the low-, moderate-, and high-priority tiers (9.3%, 28.2%, and 78.7%, respectively; Table 4), indicating an internal gradient in the model-estimated probability. However, the current tier boundaries should not be interpreted as implementation-ready decision rules. Both τ1 = 0.28 and τ2 = 0.55 were selected and evaluated using aggregated out-of-fold predictions generated from the same single-organization development data set. Consequently, the observed prevalence gradient and associated threshold-dependent performance represent internal, potentially optimistic evidence rather than an independent validation of the thresholds. Their sampling stability has not been established, and their transportability to organizations with different workforce characteristics, overtime practices, occupational health resources, or baseline fatigue prevalence remains unknown.

The observed model miscalibration further limits the direct interpretation of τ1 and τ2 as absolute probability-based decision boundaries. Notably, the observed prevalence of 78.7% in the high-priority tier, defined using τ2 = 0.55, substantially exceeded the nominal threshold, which is consistent with the calibration findings. Accordingly, prospective temporal validation, external validation in independent organizations, and local recalibration should be considered prerequisites for threshold-based implementation. Future validation should assess not only discrimination and calibration, but also threshold stability, tier volumes, sensitivity, specificity, workload implications, and potential subgroup inequities.

If validated and recalibrated, a tiered review framework could support differentiated preventive responses. Low-priority classifications might support routine monitoring and reinforcement of recovery guidance; moderate-priority classifications might prompt enhanced occupational health review of overtime exposure and recovery needs; and high-priority classifications might justify prioritized occupational health follow-up and short-term workload or schedule review, where appropriate. These examples illustrate potential future uses rather than validated intervention strategies. Such an approach would remain consistent with fatigue risk management principles emphasizing data-informed assessment, work-time monitoring, mitigation actions, and continuous review rather than reliance on fixed hours of work rules alone [16].

For Healthcare readers, the practical value of this study lies in its application to health informatics and preventive healthcare. Many occupational health systems collect working-hour records and examination data; however, these data are often underutilized for proactive workload governance. The proposed framework illustrates how routine data can be converted into explainable screening signals and, following appropriate validation, integrated into human-supervised preventive workflows. This approach may help organizations move beyond fixed overtime thresholds toward more transparent, screening-informed, and capacity-sensitive prevention strategies. The use of routine occupational health data should follow the principles of clear objectives, standardized measurements, quality control, privacy protection, and responsible data governance [17,18,19].

Any future employee-facing implementation, following prospective or external validation and local recalibration, should be subject to a separate ethical and organizational governance review and should include explicit protection for employee rights, transparency, and contestability. Employees should receive advance notice describing the purpose of the screening system, the categories of data used, the intended meaning and limitations of the model outputs, who is authorized to access individual-level results, and what follow-up actions may or may not occur. Where required by applicable law, institutional policy, or the scope of data use, appropriate consent or other authorizations should be obtained before implementation. Employees should also have access to a clearly defined process for requesting clarification, correcting inaccurate source data, obtaining human reassessment, and appealing to decisions influenced by model outputs.

Model outputs should function only as occupational health decision-support signals and should not independently determine overtime eligibility, fitness-for-work status, performance appraisal, remuneration, promotion, disciplinary action, or termination. Access to individual-level model outputs should be restricted to authorized occupational health personnel and other specifically designated reviewers under role-based access controls. Supervisors or human resources personnel should receive only the minimum information necessary to implement supportive workload or recovery measures rather than unrestricted access to individual risk scores. Final decisions should remain with authorized human reviewers who can consider contextual information not represented in the model and override model-generated recommendations when appropriate.

Governance should also include purpose limitation, data minimization, audit logging, defined data retention periods, and organizational separation between occupational health screening outputs and routine human resource performance systems. To address the risk of algorithmic bias, future implementation should include prospective subgroup monitoring of discrimination, calibration, false-positive and false-negative rates, and tier assignment by sex, job category, employment tenure, work schedule, and other relevant groups, where the sample size permits. Material subgroup disparities, calibration drift, or evidence that model outputs are being used outside their intended preventive purpose should trigger a formal review, recalibration, restriction of use, or temporary suspension. These safeguards should be documented in a model card, standard operating procedure, or occupational health governance protocol and periodically reviewed through appropriate occupational safety and health oversight, with worker representative involvement where feasible. The present study did not empirically establish the absence of algorithmic bias; therefore, these safeguards are proposed as requirements for future implementation rather than findings of the current analysis [35].

The descriptive findings also suggest plausible pathways through which subgroup differences could arise, although they should not be interpreted as evidence of bias. As shown in Table 1, work overcommitment was higher among men than women (42.9 vs. 37.9; *p* = 0.020), whereas personal fatigue and work-related fatigue did not differ significantly by sex. Because overcommitment was included only in the secondary questionnaire-enhanced specification, this finding does not demonstrate sex-related bias in the primary routine data model; however, it suggests that psychosocial exposure or reporting patterns may differ across sex groups and could influence questionnaire-enhanced models differently. Job category may similarly capture differences in workload structure, task demands, schedule flexibility, or opportunities for recovery, whereas employment tenure may be associated with cumulative exposure, occupational role, experience, or healthy-worker selection. These are hypotheses rather than demonstrated mechanisms in the present dataset, but they illustrate why comparable overall model performance does not necessarily imply comparable calibration, error rates, or tier assignment across employee subgroups.

A related implementation tension concerns worker autonomy when overtime work provides meaningful additional income. A model-generated review-priority tier should not automatically override an employee’s preference to work overtime; however, such a preference should likewise not nullify the employer’s responsibility to prevent harm associated with excessive or abnormal workloads. A proportionate approach would use an elevated screening signal to initiate a confidential occupational health review and shared decision-making, in which the worker’s preferences, current health and recovery status, available scheduling alternatives, and applicable working hour requirements are considered together. In the absence of immediate safety concerns, responses could prioritize additional recovery time, temporary schedule modifications, or other proportionate workload adjustments rather than an automatic prohibition on overtime. Any restrictions should be based on documented occupational health or safety considerations, be subject to reassessment, and provide an accessible review or appeal process. Organizations should also recognize that financial dependence on overtime may constrain meaningful choice; therefore, risk management systems should avoid shifting the economic burden of prevention solely onto individual workers.

In the Taiwanese regulatory context, these governance principles operate alongside existing occupational safety, working hours, and personal data protections. Article 6 of the Occupational Safety and Health Act requires employers to plan and adopt necessary measures to prevent ailments associated with exceptional workloads, including shift work, night work, and long working hours. Article 21 further specifies that the health examination manual should not be used for purposes other than health management, and Article 22 requires occupational health services in business entities employing 50 or more workers [36]. The Labor Standards Act establishes statutory requirements and limits for extended working hours; accordingly, model-based review-priority tiering may complement, but cannot replace, compliance with statutory working-hour protections [37]. In addition, the Personal Data Protection Act establishes data subject rights and purpose limitation requirements and places specific conditions on the collection, processing, and use of healthcare and physical examination data [38]. Together, these provisions reinforce the need for purpose-specific access, data minimization, transparent notification, correction mechanisms, and organizational separation between occupational health screening and personnel management systems. Any future employee-facing implementation would nevertheless require organization-specific legal and governance reviews rather than assuming that model validation alone establishes regulatory permissibility.

If the model is subsequently validated and considered for implementation, these protections should be embedded in formal governance artifacts that explicitly prohibit function creep beyond the model’s defined occupational health purposes. Such documentation should specify the intended and prohibited uses, authorized access, required human review and escalation procedures, data retention limits, model monitoring requirements, and mechanisms for worker feedback. These organizational controls should complement, rather than substitute, applicable occupational safety, labor, and data protection requirements. This governance framing is consistent with WHO recommendations that AI systems used in health-related contexts should be accountable, transparent, and responsive to the people affected by their use [35].

This study had several limitations. First, the retrospective design and single-organization sample limit generalizability across industries, work schedules, production systems, and organizational cultures. External validation in additional manufacturing and non-manufacturing settings is required before it can be used more broadly. Second, the observational design precludes causal inferences. These findings should not be interpreted as evidence that tier-guided actions reduce fatigue, cardiovascular risk, or safety incidents. Prospective studies are required to determine whether the use of the model improves occupational health outcomes, reduces excessive overtime exposure, or enhances recovery. Third, currently elevated work-related fatigue was operationally defined using the top quartile of the work-related fatigue subscale scores. Although this definition was suitable for the present screening-oriented analysis, its empirical cutoff may vary across populations and time periods and should not be interpreted as a universal clinical threshold. Fourth, missing data were handled using a complete case analysis. Although the available baseline characteristics did not indicate material differences between the included and excluded employees, selection bias remains possible because the excluded group was small, and information for some incomplete records was limited.

Fifth, the event-to-parameter ratio was modest, with 78 currently elevated work-related fatigue events for 25 candidate model parameters, corresponding to approximately 3.12 events per parameter. This limited information density may reduce the stability of the fitted model relationships and increase the risk of optimism, despite nested cross-validation. The reported discrimination, calibration, and feature attribution findings should therefore be interpreted as exploratory internal validation results, and substantially larger multi-site datasets are needed to assess model stability and generalizability.

Sixth, the study-specific thresholds τ1 = 0.28 and τ2 = 0.55 were selected and evaluated using aggregated out-of-fold predictions generated from the same single-organization development dataset. Although out-of-fold predictions reduce direct resubstitution bias, they do not provide independent validation of the threshold values. Consequently, the threshold-dependent classification performance, tier sizes, and observed fatigue prevalence gradients may be optimistic. The sampling stability of τ1 and τ2 was not independently assessed, and their calibration and transportability to other organizations, workforce compositions, overtime policies, or time periods remain unknown. Therefore, these thresholds should be regarded as provisional study-specific cut points rather than implementation-ready decision rules. Prospective temporal validation, external validation in independent samples, and local recalibration are prerequisites for threshold-based operational use.

Seventh, self-reported fatigue and recovery-related measures may be affected by reporting bias, timing of assessment, workplace trust, and behavioral reactivity to monitoring. Although the work-related fatigue score was excluded from the predictor set, it was used to define the study outcome, and sleep duration and weekly exercise amount were also self-reported. If employees believe that reporting fatigue, inadequate recovery, or related health concerns could reduce access to overtime or associated income, some may underreport symptoms or strategically alter questionnaire responses. More broadly, occupational reporting research has documented underreporting when workers fear adverse consequences or distrust reporting systems, providing relevant caution for employee-reported occupational health monitoring [39]. Such behavioral responses could affect outcome ascertainment and self-reported predictors and, over time, contribute to changes in response distributions, calibration drift or subgroup-differential misclassification. This retrospective study could not assess such reactivity. Therefore, future implementation should preserve confidentiality, separate occupational health screening from routine performance management, communicate clearly that self-reported information does not automatically determine employment consequences, and monitor longitudinal changes in response distributions, missingness, and model performance.

Eighth, the present sample was not sufficiently large to support a comprehensive subgroup fairness analysis. Accordingly, this study cannot establish the absence of differential discrimination, calibration errors, false-positive or false-negative rates, or disparate tier assignments across employee subgroups. In particular, subgroup analyses by sex, job category, employment tenure, and work schedule would have limited precision in the current dataset. Therefore, prospective validation in larger and more diverse samples should include prespecified fairness analyses before implementation, followed by ongoing subgroup performance monitoring after deployment.

Future research should prioritize prospective temporal and external validation in independent organizations before implementation. These studies should explicitly assess the stability and transportability of τ1 and τ2, including discrimination, calibration, sensitivity, specificity, tier volumes, and occupational health workload. Subgroup performance should be assessed by sex, job category, employment tenure, and work schedule using subgroup-specific discrimination, calibration, false-positive and false-negative rates, and tier-assignment distributions to identify potential inequities or disparate impact. Where sample sizes permit, differences in model performance and threshold consequences should be quantified with appropriate uncertainty estimates rather than interpreted solely from point estimates.

Calibration and tier volumes should be monitored over time to detect dataset shifts as staffing patterns, overtime policies, and production demands change. Future studies should evaluate whether integrating additional recovery indicators, such as shift schedules, rest intervals, sleep quality, workload variability, and employee-reported recovery, improves prediction and governance utility. Finally, implementation research should examine employee understanding of the system, adequacy of advance notification, perceived voluntariness and trust, acceptance of data use, accessibility of reassessment and appeal mechanisms, whether awareness of screening alters symptom reporting or questionnaire response patterns over time, supervisor and occupational health use, workflow integration, and safeguards against punitive or unauthorized secondary use.

In summary, routinely collected working-hour, recovery-related, and occupational health examination data can be integrated into an explainable framework for the concurrent screening of currently elevated work-related fatigue. XGBoost showed better internal validation performance than logistic regression, while the contributions of cumulative overtime, recovery-related variables, and routine physiological indicators supported an exposure–recovery–vulnerability interpretation. However, the modest information density, systematic miscalibration, and study-specific derivation of probability thresholds require independent prospective and external validation before implementation. Any future employee-facing use would also need to operate within the applicable occupational safety, working-hour, and data protection requirements.

## 5. Conclusions

Routinely collected working-hour records and occupational health examination data can be integrated into an explainable, probability-based concurrent screening framework to identify employees with currently elevated work-related fatigue and support preventive overtime reviews among manufacturing workers. In internal validation, the XGBoost model showed better discrimination and overall probabilistic accuracy than logistic regression and enabled a provisional review-priority tiering framework intended to align review intensity with occupational health capacity. Medium-term cumulative overtime, together with routinely measured indicators of vulnerability and recovery, contributed meaningfully to the model-estimated probability of currently elevated fatigue, supporting the relevance of cumulative exposure and recovery-related strain in preventive healthcare in the workplace.

However, calibration diagnostics indicated that the predicted probabilities were not sufficiently calibrated for direct threshold-based applications. Moreover, τ1 and τ2 were derived and evaluated using the same single-organization dataset. Therefore, these thresholds should be regarded as provisional, study-specific cut points rather than fixed operational decision boundaries, and their stability and transportability remain unknown. Prospective temporal validation, external validation in independent organizations, and local recalibration are required before threshold-based implementation.

## Figures and Tables

**Table 1:** Descriptive fatigue and overcommitment scores by sex and average daily working hours.

		Personal Fatigue, Mean (SD)	Work-Related Fatigue, Mean (SD)	Work Overcommitment, Mean (SD)
By sex	Male (*n* = 205)	34.5 (15.8)	30.8 (16.7)	42.9 (17.5)
	Female (*n* = 109)	37.8 (17.5)	33.3 (17.4)	37.9 (18.3)
	*p*-value	0.104	0.209	0.020
By average daily working hours	<8 h (*n* = 47)	33.7 (17.3)	28.5 (16.9)	37.2 (16.5)
	8–10 h (*n* = 250)	36.1 (16.3)	32.3 (17.0)	41.3 (17.7)
	≥10 h (*n* = 17)	34.1 (18.5)	30.9 (16.4)	50.6 (16.6)
	*p*-value	0.626	0.368	0.034

Notes: Subscale scores were calculated as the mean of five 5-point Likert items and subsequently linearly transformed to a 0–100 scale, with higher scores indicating greater fatigue or overcommitment. *p*-values were derived from two-sample *t*-tests for comparisons by sex and one-way analysis of variance for comparisons across the working-hour groups.

**Table 2:** Overtime exposure indicators and threshold exceedance in the fatigue group.

Overtime Indicator	Overall (n = 314)	Non-High Fatigue (n = 236)	High Fatigue (n = 78)	p-Value
Monthly overtime, month −1 (hours)	24 (12–42)	22 (10–38)	33 (18–55)	<0.001
Cumulative overtime, months −2 to −6 (hours)	125 (70–195)	112 (60–175)	170 (105–245)	<0.001
Cumulative overtime, months −1 to −7 (hours)	175 (95–265)	160 (85–240)	235 (140–320)	<0.001
Exceeded ≥37 overtime hours/month	92 (29.3)	61 (25.8)	31 (39.7)	0.022
Exceeded ≥72 overtime hours/month	24 (7.6)	14 (5.9)	10 (12.8)	0.081
Exceeded ≥92 overtime hours/month	10 (3.2)	5 (2.1)	5 (6.4)	0.128

Notes: Values are reported as median (IQR) for overtime hours and *n* (%) for threshold exceedance. Group differences in continuous overtime variables were assessed using the Mann–Whitney U-test. Threshold-exceedance indicators were compared using Fisher’s exact test because of the small cell counts at higher overtime thresholds. Threshold exceedance was defined as exceeding the specified threshold in any month from month −1 to month −7 relative to the index assessment. These thresholds correspond to the organization’s internal overtime monitoring system and are used for a prevention-oriented workload review.

**Table 3:** Internal validation performance of the routine data specification.

Performance Metric	Logistic Regression	XGBoost
AUC (95% CI)	0.68 (0.62–0.74)	0.77 (0.71–0.83)
Brier score	0.17	0.14
Sensitivity (recall)	0.67	0.78
Specificity	0.69	0.70
Accuracy	0.68	0.72
Precision	0.42	0.46
F1 score	0.51	0.58

Notes: Model performance was evaluated using stratified five-fold cross-validation with aggregated out-of-fold predictions. The 95% confidence intervals for the AUC were calculated using 2000 bootstrap resamples of the out-of-fold predictions. The classification metrics were computed using a study-specific provisional threshold of τ1 = 0.28. Because this threshold was selected and evaluated using the same dataset, the reported classification metrics should be regarded as exploratory and internal estimates. For XGBoost, the threshold τ1 classified the moderate- and high-priority tiers as screen positive and the low-priority tier as screen negative. The paired AUC difference between XGBoost and logistic regression was assessed using the DeLong test.

**Table 4:** Provisional study-specific review-priority tiers and observed prevalence of currently elevated work-related fatigue based on extreme gradient boosting out-of-fold predicted probabilities.

Review-Priority Tier	Estimated Probability Range	n (%)	Currently Elevated Fatigue, n	Non-High Fatigue, n	Observed Prevalence of Elevated Fatigue, n/N (%)	Intended Review Response
Low priority	*p* < 0.28	182 (58.0)	17	165	17/182 (9.3)	Routine monitoring and recovery guidance are provided.
Moderate priority	0.28 ≤ *p* < 0.55	85 (27.1)	24	61	24/85 (28.2)	Enhanced occupational health review of overtime exposure and recovery needs.
High priority	*p* ≥ 0.55	47 (15.0)	37	10	37/47 (78.7)	Prioritized occupational health follow-up and short-term schedule adjustments when appropriate.

Notes: Thresholds τ1 = 0.28 and τ2 = 0.55 were selected and evaluated using aggregated out-of-fold predictions generated from the same development dataset. The resulting tiers were provisional and study-specific. Their stability and transportability to other organizations, populations, or time periods remain unknown, and prospective or external validation with local recalibration is required before their operational implementation.

## Data Availability

The data are not publicly available due to employee privacy, occupational health confidentiality, and organizational data protection. De-identified analytic data may be made available from the corresponding author upon reasonable request, with approval from the responsible occupational health governance body, where permitted by applicable regulations and institutional policy.
